# Comment on “Dual-Band Perfect Metamaterial Absorber Based on an Asymmetric H-Shaped Structure for Terahertz Waves [Materials] (2018) [2193; https://doi.org/10.3390/ma11112193]”

**DOI:** 10.3390/ma12233914

**Published:** 2019-11-27

**Authors:** Fahad Ahmed, Afzal Ahmed, Tania Tamoor, Tayyab Hassan

**Affiliations:** Research Institute for Microwave and Millimeter-wave Studies, National University of Sciences and Technology, Islamabad-44000, Pakistan; aahmed.phdee17seecs@seecs.edu.pk (A.A.); ttamoor.msee19seecs@seecs.edu.pk (T.T.); thassan.msee16seecs@seecs.edu.pk (T.H.)

In a recent publication, Lu et al. [1] proposed a dual-band perfect metamaterial absorber based on an asymmetric H-shaped structure for terahertz waves. The authors claimed 99.95% and 99.91% absorptions at two resonance frequencies of 4.73 THz and 5 THz, respectively. In this paper, we demonstrate that the authors erroneously interpreted cross-polarizer as a dual-band absorber. By including both polarized reflection coefficients (co- and cross-), the absorption decreases from 99.91% to 21% at 5 THz. However, absorption remains at 99.95% at 4.73 THz but disappears when FR-4 (lossy) substrate is used. It is worth mentioning that the authors designed the absorber on FR-4 (loss-free) substrate, which is commonly used in ideal conditions. We analyzed the results for both lossy and loss-free FR-4 substrate. Additionally, the polarization conversion ratio (PCR) is approaching 85% at a resonance frequency of 5 THz, which shows that the proposed metasurface is a cross-polarizer rather than an absorber.

## 1. Analysis and Results

In the last few years, metasurface-based absorbers have been extensively studied because of their ease of fabrication and low cost. However, the absorbers present in the literature are complex, narrowband, and multilayer structures. It is still challenging to obtain broadband absorption by using a single-layer periodic structure [2,3,4]. Many such single-layer periodic structures are found in literature, which researchers claim as absorbers [5,6,7,8,9], but they are actually cross-polarizers [10,11,12,13,14]. The authors of these papers concentrated merely on the co-polarized reflection coefficient of the incident wave and falsely deduced their designs as perfect broadband absorbers. To achieve actual absorptivity, both polarized reflection coefficients (co- and cross-) should be included in the absorption calculations.

**Figure 1:**
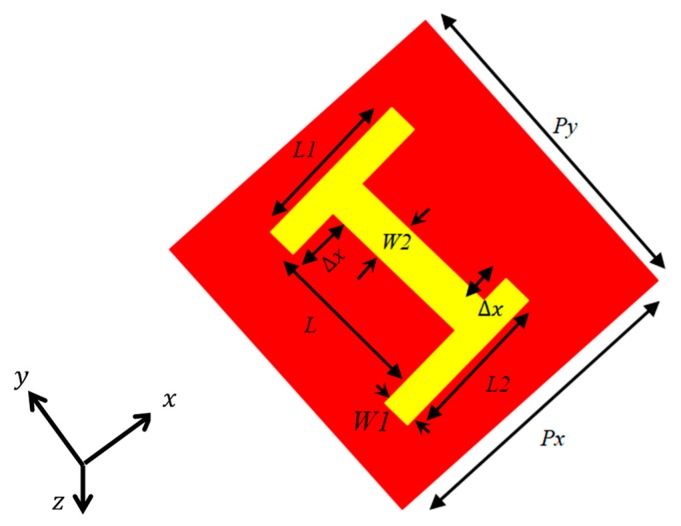
Top view of unit cell. The geometric parameters are the following: ∆x = 9 µm, L = 28 µm, L1 = L2 = 27 µm, W1 = 4 µm, W2 = 5 µm, P*_x_* = P*_y_* = 40 µm.

When a linearly polarized EM wave is incident upon a surface, then its absorptivity *A*(*ω*) is given as follows:

(1) $$A\left(\omega \right)=1-{\left|R_{yy}\right|}^{2}-{\left|R_{xy}\right|}^{2}-{\left|T_{yy}\right|}^{2}-{\left|T_{xy}\right|}^{2},$$

where ${\left|R_{yy}\right|}^{2}$, ${\left|R_{xy}\right|}^{2}$, ${\left|T_{yy}\right|}^{2}\;\mathrm{and}\;{\left|T_{xy}\right|}^{2}$ are co- and cross-polarized reflection and transmission coefficients, respectively. To stop any kind of transmission (${\left|T_{yy}\right|}^{2}-{\left|T_{xy}\right|}^{2})$, the backside of the metasurface is grounded with copper and hence T is equal to zero. For this comment, we investigated a metasurface based reflective single-layer terahertz “absorber” proposed by Lu et al. [1]. The proposed “absorber” consisted of a dielectric layer of FR4 substrate with loss tangent *δ* = 0 (loss free) and $\varepsilon_{r}$ = 4.3. Shown in Figure 1, the top side of the substrate consisted of an H-shaped structure (asymmetric), while the bottom layer consisted of a metallic ground plane. The metal of both layers was copper with a thickness of 0.036 μm and electric conductivity of 4.58 × 10^7^ S/m.

**Figure 2:**
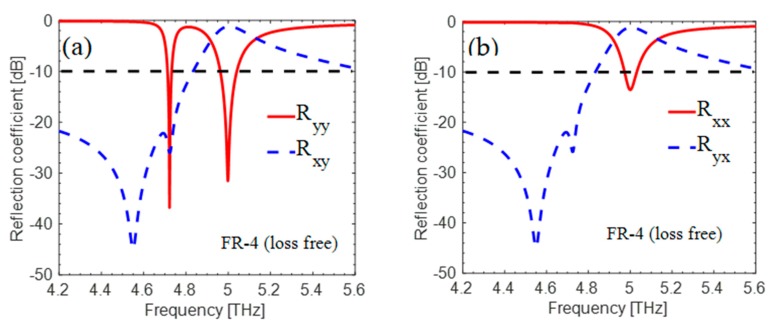
(**a**,**b**) show simulated cross- and co-polarized reflections, respectively. Here, $R_{\mathrm{yy}}$ and $R_{\mathrm{xx}}$ are co-components and $R_{\mathrm{xy}}$ and $R_{\mathrm{yx}}$ are cross-components for *y* and *x*-polarized incident waves, respectively.

The proposed “absorber” was simulated numerically in CST Microwave Studio Software by using unit cell boundary conditions in the direction of the *x*- and *y*-axes and Floquet ports in the *z*-direction. The key role of cross-components for both polarized incident waves (x and y) is shown in Figure 2a,b, respectively. The original issue is with the cross-polarized reflection coefficient ${\left|R_{xy}\right|}^{2}$, which most authors neglect when simulating their absorbers. The authors of the paper also neglected the cross-polarized reflection coefficient as depicted in Figure 2, and the incident EM wave converted into its cross-counterpart at 5 THz.

**Figure 3:**
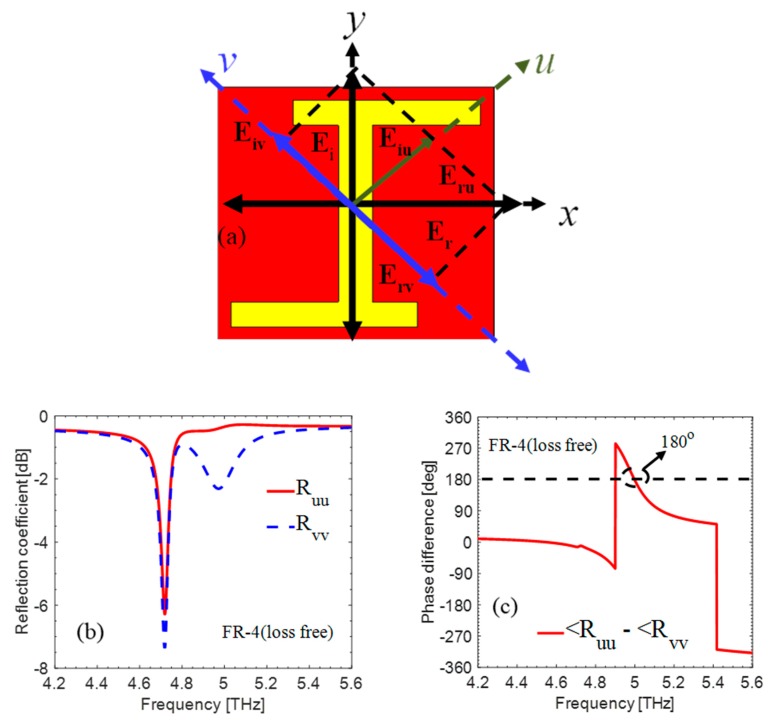
(**a**) Top view of unit cell depicting the decomposition of *x*- and *y*-axes. (**b**) Magnitude of the *v*- and *u*-polarized components. (**c**) Phase difference between *u*- and *v*-components.

To analyze the mechanism of polarization conversion, we decomposed the incident electric field into *u* and *v* components at ±45° with reference to x- and y-component as depicted in Figure 3a. The simulation is performed by determining u- and v- polarized incident field. In analysis, we investigated that the reported metasurface is also polarization-dependent at 4.73 THz. The reflected EM wave is transformed into its cross-component when the criteria of magnitude |R_uu_| = |R_vv_| and phase difference ±180° are fulfilled at 5 THz as shown in Figure 3b,c. Due to dielectric and conduction losses, the magnitude of |R_vv_| is less than or equal to |R_uu_| at some frequencies.

**Figure 4:**
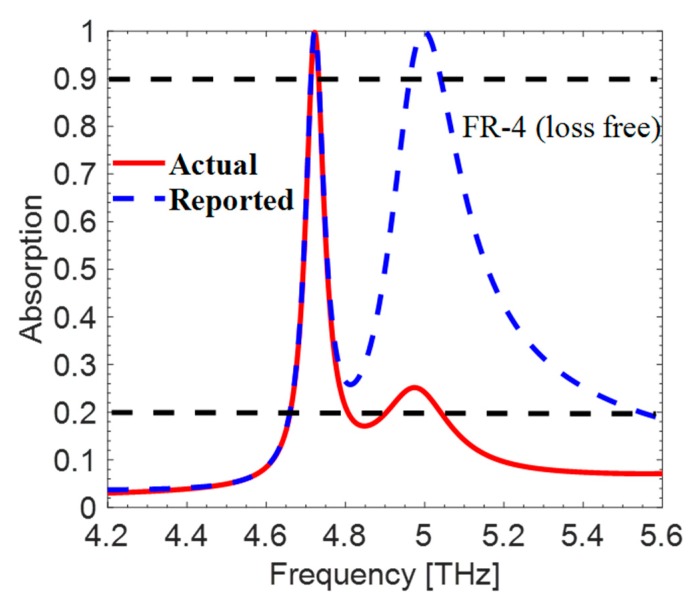
The reported and actual absorption comparison of the proposed metasurface.

When the cross-polarized reflection coefficient of the incident wave is included in Equation (1), the absorptivity becomes much less, as is shown in Figure 4. The actual absorptivity is almost 21% at the resonance frequency of 5 THz, but the author claimed 99.91%.

Lastly, given the cross-polarization conversion property of a metasurface, it is intriguing to study the PCR (polarization conversion ratio), that is:

(2) $$\mathrm{PCR}=\frac{{\left|Rxy\right|}^{2}}{{\left|Rxy\right|}^{2}\;+{\left|Ryy\right|}^{2}}.$$

**Figure 5:**
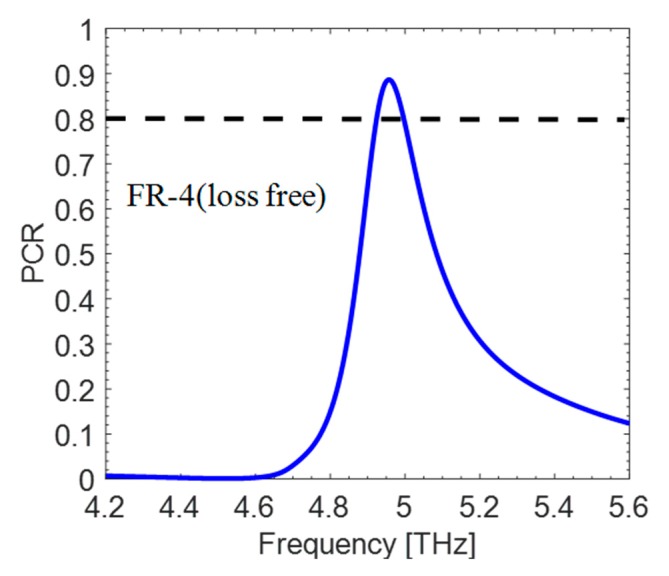
The calculated PCR for the structure.

Figure 5 shows that the designed structure has a PCR of 80% at a resonance frequency of 5 THz. Looking at the high PCR, we can say that the metasurface under observation is an efficient broadband cross-polarizer and not a perfect absorber.

**Figure 6:**
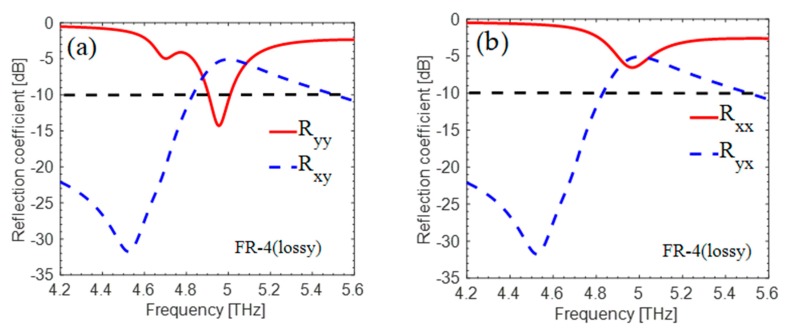
(**a**,**b**) shows simulated cross- and co-polarized reflections. Here, $R_{\mathrm{yy}}$ and $R_{\mathrm{xx}}$ are co-components and $R_{\mathrm{xy}}$ and $R_{\mathrm{yx}}$ are cross-components for *y* and *x*-polarized incident waves, respectively, for FR-4 lossy substrate.

Simulations for FR-4 lossy substrate were also performed. The absorption at 4.73 THz disappeared, as is shown in Figure 6, and the structure started behaving as a cross-polarizer. The authors mistakenly chose the ideal substrate that is not available commercially.

## 2. Summary

The authors in [1] proposed a design and mistakenly reported it as a dual-band absorber. We investigated this matter and argue here that the design is deficient of absorption properties. The reported structure behaved as an efficient cross-polarizer at 5 THz. Hence, the use of this design is restricted only to applications where cross-polarizing operations are required at 5 THz.
